# An interview with Eiji Tanaka

**DOI:** 10.1590/2176-9451.20.1.030-039.int

**Published:** 2015

First of all, I would like to express my gratitude to Dental Press Journal of Orthodontics for the opportunity to conduct this interview with professor Eiji Tanaka, a brilliant scientist and dear friend of mine. Eiji Sensei, as he is affectionately known in Japan, is a simple and very charismatic man. I consider him a fatigueless scientist and a talented supporter who encourages others to develop their skills. I never forget his favorite quote: "No pain, no gain". We were once watching a show about the therapeutic use of ultrasound to recover David Beckham's bone lesion during the World Cup 2006, when Eiji said: "Why nobody uses ultrasound to recover dental tissues?" From that point onwards, he became the pioneer of studies on the topic in Orthodontics, particularly with regards to root resorption. Professor Tanaka holds a DDS and PhD degree in Orthodontics from Osaka University. He is a former associate professor from Hiroshima University and has been the head of the Department of Orthodontics and Facial Orthopedics at The University of Tokushima since 2008. He is also a member of the Journal of Biomechanics and Journal of Dental Research editorial board, and associate editor of the Annals of Biomedical Engineering. Moreover, professor Tanaka has more than 180 articles published in renowned international periodicals. His studies mainly focus on the following: Temporomandibular joint (TMJ) biomechanics, therapeutic application of low-intensity ultrasound in Tissue Engineering and development of genetic approaches towards treatment of atrophic and degenerative TMJ muscle disease.

Gostaria de expressar minha gratidão ao *Dental Press Journal of Orthodontics*, pela oportunidade de conduzir essa entrevista com o professor Eiji Tanaka, um grande amigo e um brilhante cientista. Eiji Sensei, como carinhosamente é chamado no Japão, é uma pessoa simples e muito carismática. Considero-o um incansável cientista e um talentoso incentivador de habilidades. Não me esqueço de sua frase predileta "*No pain, no gain*". Certa vez, assistindo a um programa sobre o uso de ultrassom terapêutico na recuperação de uma lesão óssea sofrida por David Beckham, na Copa do Mundo de 2006, Eiji disse: "Por que ninguém usa o ultrassom para reparação de tecidos dentários?". A partir daí, tornou-se precursor no estudo desse tópico em Ortodontia, sobretudo no tocante à reabsorção radicular. O professor Tanaka é graduado em Odontologia e PhD em Ortodontia pela Universidade de Osaka. Foi professor associado na Universidade de Hiroshima, e desde 2008 ocupa o cargo de chefe do departamento de Ortodontia e Ortopedia Facial da Universidade de Tokushima, no Japão. Faz parte do corpo editorial do *Journal of Biomechanics* e do *Journal of Dental Research*, e é editor associado do *Annals of Biomedical Engeneering*. Possui mais de 180 artigos publicados em relevantes periódicos internacionais. Seus estudos contemplam, principalmente, os temas: biomecânica da articulação temporomandibular (ATM), aplicação do ultrassom terapêutico de baixa intensidade em engenharia tecidual, e desenvolvimento de abordagens genéticas para o tratamento de doenças musculares atróficas e degenerativas da ATM.

Emanuel Braga Rêgo

## Professor, I had the opportunity to be advised by you during my PhD course in Hiroshima University and I was amazed by the research environment. It was very pleasant to use modern labs, research structure and, of course, to have access to funding for experiments. Back to Brazil, I could experience some difficulties in performing great impact science. Could you please explain how does governmental funding work for the orthodontic sciences in Japan? Emanuel Braga Rêgo 

In Japan, Grants-in-Aid for Scientific Research is established within the Japan Society for the Promotion of Science (JSPS) from the Ministry of Education, Culture, Sports, Science and Technology (MEXT; Monbu Kagakusho). Grants-in-Aid is awarded to promote creative and pioneering research across a wide spectrum field including Orthodontics and Dentistry, ranging from the humanities and social sciences to the natural sciences. Grants are awarded to projects organized by individual researchers or research groups at Japanese Universities or Research Institutes engaged in basic research, particularly research in critical fields attuned to advanced research trends. Every year we generally apply for Grants-in-Aid for Science Research, and can perform research experiments and obtain research results provided our research project application is accepted. Furthermore, I routinely apply for the Japanese Science and Technology Agency in Japan (JST), an agency that provides infrastructure for the entire process from creation of knowledge to the return to society. JST cooperates in planning research and developing strategies for the creation of innovation, in addition to promoting the creation of innovation. Among several competitive funding programs, we also apply for A-Step (Adaptable & Seamless Technology Transfer Program through Target-driven R&D). This program supports collaborative industry-academia R&D based on the results of high-quality basic research to ensure that the benefits of research are passed onto Japanese society, which matches up to my research standpoint. In the last five years, we have obtained research grants from JSPS and JST of more than $50,000 per year.

## Professor Tanaka, during my stay in Japan, I noticed that private companies are great enthusiasts of research performed at Universities. I honestly think that the Japanese model could be adopted in Brazil in order to improve research conditions at Universities and Institutes. What is your opinion about that? Could you please give a brief explanation on private sponsoring for research in Japan? Emanuel Braga Rêgo

Although I know nothing about the research conditions in Brazilian Universities and Institutes, your idea that the Japanese model could be adopted in Brazil is, in my opinion, great and reliable. In Japan, there are many private companies wanting to establish research collaboration with Universities and Institutes from all around the world, as important innovations and development have been made by means of partnerships established between industry-academia. However, it is considered difficult for even us to get a chance to establish such a good relationship with a private company. One possible way to build a good relationship with a private company is to attend professional small meetings and conferences taken place all around the world and try to establish fruitful communication with the company staff, introducing your research idea and project and, whenever possible, with preliminary data.

## Professor, what are the current topics being researched at the University of Tokushima and what do you think will be the "hot spot" in Orthodontics research in the near future? Emanuel Braga Rêgo

Now I am focusing on highly efficient targeted mutagenesis in one-cell mouse embryos mediated by new gene-targeting technologies, transcription activator-like effector nuclease (TALEN) and the clustered, regularly interspaced, short palindromic repeat (CRISPR)/CRISPR-associated protein (Cas) system. We have recently published a paper in the Scientific Reports (Nature Publishing Group). Since the establishment of embryonic stem (ES) cell lines, gene targeting combined with homologous recombination has aided our understanding of the functions of various genes. However, the ES cell technique is inefficient, time-consuming and labor-intensive. The newly developed technologies, TALEN and CRISPR/Cas systems, enable researchers to induce site-specific mutations in various species for which ES cells have not been established. We demonstrate the high efficiency of TALEN (with efficiency of up to 50%) and CRISPR/Cas (with efficiency of 90%) systems for producing mutant mice for the Fgf10 gene by RNA microinjection in one-cell mouse embryos. We also confirm the germline transmission of TALEN-induced mutations. Our results provide evidence that the TALEN and CRISPR/Cas systems are an excellent tool to accelerate functional genomic research in mice. Right now, by means of these technologies, we have been developing many specific gene knockout mice, which are very useful to develop a new treatment remedy for gene-related diseases.

Regarding the "hot spot" in orthodontic research, growing evidence suggests that miniscrew anchorage greatly expands the limit of clinical orthodontics. Even without patient compliance, miniscrews can provide stationary anchorage for various tooth movements and even make it possible to move the tooth in directions which have been rendered impossible with traditional orthodontic mechanics. Nowadays miniscrew anchorage has been used by numerous orthodontists. On the other hand, the clinical use of miniscrew anchorage involves some risks. Screw fracture might be one of the most undesirable side effects in clinical use of miniscrew anchorage, which occurs not only in placement, but also during removal. A recent systematic review revealed that the overall success rate of miniscrews was 86.5%, which is significantly lower than that of dental implants for prosthetic restorations. A lot of factors are suggested to relate with screw failure, but screw-root and screw-mandible proximity are considered as two common factors. Therefore, we would like to develop a new miniscrew implant system with higher success rates in the near future, which would provide us with the clinical recommendations to reduce risks and concerns.

## Root resorption is certainly one of the great challenges in Orthodontics. Approaches have been suggested to deal with orthodontic-induced root loss, but a well accepted therapy has not yet been established. In this context, low-intensity pulsed ultrasound (LIPUS) seems to be a promising field and I am very excited with the results published by Professor Tanaka. Could you please briefly explain the biomolecular process involved in LIPUS-induced root protection and healing? André Wilson Machado

I appreciate your interest in our research about LIPUS. We have started doing research using LIPUS in 2006 with my colleagues, Dr. Diego Dalla-Bona, Dr. Emanuel Rêgo from Brazil and Dr. Toshihiro Inubushi.

In general, after root resorption, the roots are repaired by cementoblasts. Cementoblast adhesion, activation and subsequent root repair are thought to be associated with temporospatial expression and maturation of various extracellular matrix proteins. Severe root resorption has been usually associated with aggressive orthodontic tooth movement, but is often noted without an explanation for the cause (idiopathic root resorption). Even now treatment remedies for protection against root resorption and/or for repair of absorbed roots are limited.

With optimal force application, bone resorption is induced during tooth movement, although tooth root resorption is generally much less done so. That is to say, while many osteoclasts appear on the bone surface and induce bone resorption, cementoclasts are not derived. It is generally accepted that cementoclasts and osteoclasts are derived from mononuclear hematopoietic progenitor cells and share many characteristics, such as high TRAP staining and hard tissue resorbing activity. In the process of osteoclasts differentiation and maturation, osteoblasts induce RANKL and OPG plays an important role. Furthermore, MCP-1, MIP-2 and RANTES are chemokines which take part in mechanically induced bone remodeling. Likewise, cementoclasts are deeply involved in odontoclast maturation processes during root resorption. Cementoblasts share many molecular properties with osteoblasts, including type I collagen and non-collagenous proteins, such as osteocalcin, osteopontin and bone sialoprotein. Recently, it has been reported that cementoblasts have specific gene expression, such as cementum-derived attachment protein (CAP), cementum-derived protein (CP-23), F-spondin and so on. Therefore, cementoblasts are considered to be a unique cell type when compared to osteoblasts. We showed different osteo/cementoclastic gene expression in osteoblasts and cementoblasts. Berry et al^1^ also reported that cementoblasts show high OPG mRNA expression. This may indicate that cementoblasts prevent root resorption during orthodontic tooth movement. However, in heavy force-induced severe root resorption, not only osteoclasts, but also cementoclasts were activated on the root surface. It has been reported that LIPUS exposure promotes bone remodeling by means of increasing RANKL, MCP-1, MIP-1β and MIP-2 mRNA expression in osteoblasts. Our results also demonstrated that LIPUS enhanced RANKL mRNA expression in osteoblasts and cementoblasts, while LIPUS increased OPG mRNA expression only in cementoblasts.

## Despite yielding great results, the LIPUS technology has not been fully used by orthodontists. Do you believe that additional research is still needed? When do you think LIPUS will be universally available on the market? André Wilson Machado

LIPUS has been extensively used as a therapeutic, operative and diagnostic tool in Medicine, but not in Dentistry. It has been considered that the pulsed frequency of ultrasound results in mechanical vibration and stimulates tissues. Many supportive studies have demonstrated that LIPUS can promote bone repair and regeneration, accelerate bone fracture healing and enhance osteogenesis at the distraction site. Recently, the effect of ultrasound on soft tissues has been paid much attention to. It has been reported that LIPUS promoted cell proliferation in fibroblasts and myoblasts and increased the mRNA level of connective tissue growth factor CCN2/CTGF in gingival epithelial cells. Furthermore, LIPUS reduces inflammation and promotes regeneration in various injured soft tissues, such as synovitis of the knee joint, collateral ligament injury and injured skeletal muscle. Thus, ultrasound may be considered a therapy with clinical potential to be used in the reduction of soft tissue healing time in oral and maxillofacial regions.

Importantly, LIPUS has no thermal and destructive effects and distinguishes itself by being non-invasive and easy to apply. Based on previous experimental results, we now have started to make clinical use of LIPUS as a preventive therapy for root resorption. Of course, we have obtained permission to perform clinical trials with LIPUS from the Institutional Review Board of the Tokushima University Hospital. Whenever we are able collect more than 50 data from orthodontic patients, we will propose some patents. 

## What are the next steps regarding LIPUS technology and research? André Wilson Machado

We have investigated the effect of LIPUS on synovial membrane cells and synovium metabolism in rheumatoid arthritis (RA) and OA patients in order to evaluate the effectiveness of LIPUS treatment against synovitis in RA and OA joints. Synovial hyperplasia is a major pathophysiologic feature of RA and appears to be associated with proinflammatory cytokines, notably TNF-α and IL-1β. Therefore, the importance of synovitis in RA joints has been increasingly recognized, particularly at early stages of the disease. Furthermore, synovial fibroblasts in the synovial intimal lining play a key role in producing cytokines and proteases. Since targeting synovial fibroblasts may improve clinical outcomes in inflammatory arthritis, it is thought that the control of metabolism of synovial fibroblasts is an important consideration for treatment strategies. We have previously demonstrated that the increased expression of Cox-2 in IL-1β-stimulated synovial membrane cells was significantly inhibited by LIPUS exposure *in vitro*. In addition, we showed that LIPUS affected apoptosis and proliferation of rabbit synovial cells, HIG-82, and the expression of Cox-2 in the knee joints of MRL/lpr mice was markedly reduced by daily LIPUS exposure. Therefore, it can be hypothesized that the inhibition of Cox-2 expression by LIPUS exposure inhibits cell proliferation in synovial tissue as a secondary effect. In our recent study, LIPUS exposure increased phosphorylation of FAK, JNK, ERK, and p38, but the phosphorylation was inhibited by FAK phosphorylation inhibitor, thereby indicating that LIPUS exposure might be involved in cell apoptosis and survival of synovial membrane cells via integrin/FAK/MAPK pathway. All in all, LIPUS stimulation may be a better medical treatment for joint inflammatory diseases, such as OA and RA.

## What are the major contributions of biomedical technology, such as CBCT and MRI, to the diagnosis and treatment approach of temporomandibular joint disorders (TMD)? Clarice Nishio

It is a great honor to be interviewed by Dr. Clarice Nishio. The major contribution of biomedical technology, CT and MRI, for TMD diagnosis and treatment planning is being visible to bone morphology and disc structure. CT provides important information about bone structure. Normal condyle shows uniform cortical bone thickness, while osteoarthritic condyle shows bone deformation, such as erosion, destructive, sclerosis and osteophyte. Computer graphic technique has been markedly developed and has enabled us to reconstruct TMJ three-dimensionally. As a result, we can easily make a three-dimensional TMJ model based on CT DICOM data. This is beneficial for us to understand three-dimensional structural and pathological characteristics in detail. However, CT does not provide spatial and structural information of the TMJ disc. For this reason, MR images are taken in cases of radiologic evidence of the presence or absence of TMJ internal derangement, as well as in cases of joint effusion, articular surface irregularities and alterations of bone marrow in the mandibular condyle. Even after CT and MRI examination, should dysfunctional bone remodeling and spatial and structural changes of the disc not be detected, the patient will be suspected to have myofacial pain-dysfunction (MPD) syndrome, capsule-ligament disorders or psychological problems.

**Figure 1 -:**
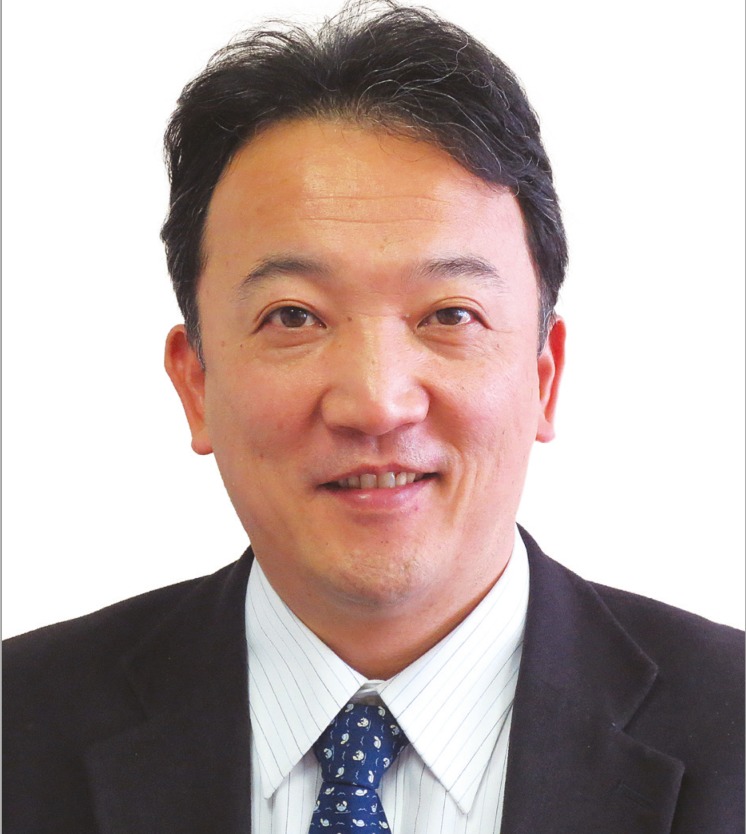
LIPUS (BR Sonic Pro, ITO Corp., Tokyo, Japan) used as preventive therapy for root resorption.

## What are the current status and the future of fundamental and clinical research conducted to improve the diagnostic and treatment modalities of patients who suffer from TMD? Clarice Nishio

Based on numerous clinical and fundamental research articles, including our papers, we have summarized the concept of mandibular condylar cartilage degradation. Under normal conditions, functional loading in TMJ is essential to functional bone remodeling and metabolism. This loading is absolutely necessary for growth, development and maintenance of the TMJ. However, if loading is excessive or abnormal, or if the host adaptive capacity decreases due to aging and systematic disease, functional overloading can facilitate hypoxia in the TMJ, which mediates the destructive processes associated with osteoarthritis (OA) as an autocrine factor. Vascular endothelial growth factor (VEGF) induction in OA-cartilage by functional overloading is linked to activation of hypoxia inducing factor-1 (HIF-1), which leads to hypoxia in the joint tissue. Furthermore, VEGF regulates the production of MMPs and TIMPs, both of which are among the effectors of extracellular matrix remodeling. Overloading also causes collapse of joint lubrication as a result of hyaluronic acid degradation by free radicals. The regulation of hyaluronic acid production is controlled by various pro-inflammatory cytokines. As a result, TMJ overloading is one of the key roles in OA onset and progression. In the future, we would like to try to identify the relationship between TMJ overloading and OA pathology, using HIF-1 and/or VEGF knockout animal model. In addition, we would like to develop an innovative strategy as a new orthodontic treatment remedy for TMJ-OA.

**Figure 2 -:**
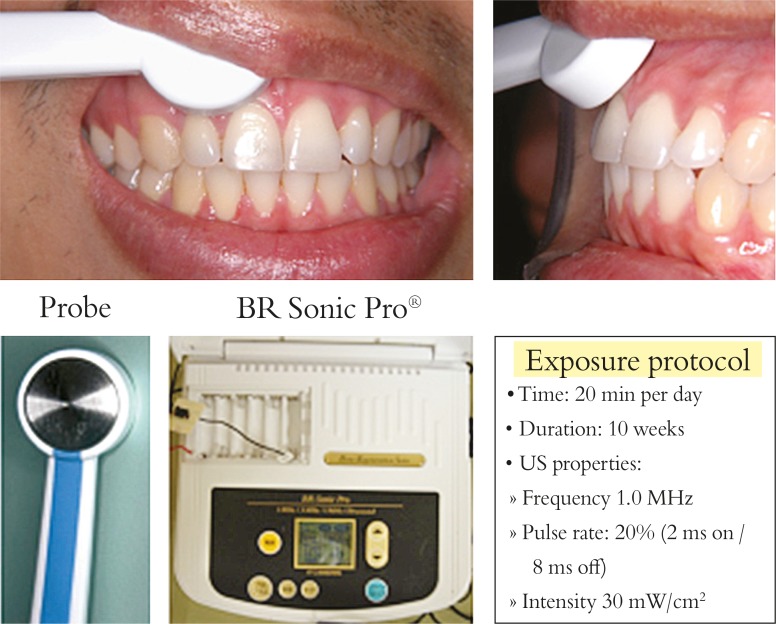
Schematic illustration of mechanisms of signal transduction pathways enhanced by LIPUS (Low intensity Pulsed Ultrasound). LIPUS may regulate synovial cell metabolism via integrin/FAK/MAPK pathway particularly.

## Based on your extensive orthodontic clinical experience, what do you consider to be a major challenge when treating patients with TMD? Clarice Nishio

Management of TMJ-OA is divided into non-invasive, minimally invasive, invasive or surgical modalities, and salvage modalities in end-stage disease. The decision to surgically manage any TMJ arthritic condition must be based on evaluation of patient's response to non-invasive management, patient's mandibular form and function, and the effect the condition has on patient's quality of life. Orthognatic surgery is included in invasive surgical modalities. However, is osteotomy successful for the management of TMJ-OA? Many reports have been published in which orthognathic surgery may not be successful for management of TMJ-OA. These reports demonstrate that treatment outcomes after orthognathic surgery depend on presurgical TMJ conditions. Patients with active TMJ disease and either concomitant or resultant maxillofacial skeletal discrepancies treated only by means of orthognathic surgery often have poor outcomes and significant relapse. This implies that patients with presurgical TMJ symptoms requiring mandibular advancement appear to be at increased risk of condylar resorption. Furthermore, degenerative and osteolytic changes make the TMJ components highly susceptible to failure under the new functional loading resulting from orthognathic surgical repositioning of the maxillofacial skeleton. 

It is true that morphological collapse of the joint component by TMJ-OA induces a decrease in ramus height, thereby leading to clockwise rotation of the mandible and anterior open bite. These characteristics appear to cause TMJ overloading. Results of finite element model analysis reveal that open bite can induce larger stress in the TMJ compared with normal occlusion. Furthermore, clockwise rotation of the mandible, which is a main characteristic of skeletal open bite, leads to a synergistic increase of TMJ stress during clenching. This indicates that improvements in mandibular clockwise rotation may be essential for the treatment of acquired open bite with TMJ-OA, resulting in the reduction of TMJ overloading.

Therefore, we recommend orthodontic treatment for patients with OA-associated condylar resorption and either concomitant or resultant maxillofacial skeletal discrepancies, such as mandibular retrusion and anterior open bite with molar intrusion, as it not only has a beneficial effect on esthetic appearance and occlusion, but also results in TMJ improvement. As a result of counterclockwise rotation of the mandible caused by molar intrusion, the condyle is repositioned, and functional adaptation in circumoral musculature can be achieved. Treatment with implant anchorage for molar intrusion might become a new therapeutic approach for anterior open bite patients with TMD. In our experience, long-term follow-ups (at least 5 years) after orthodontic treatment confirmed no or minimal relapse of mandibular clockwise rotation, anterior open bite and recurrence of TMD symptoms. Therefore, it is essential to understand the pathogenesis of TMJ-OA and current clinical treatment modalities in order to develop a "good as new" treatment remedy for TMJ-OA, including the orthodontic approach.

**Figure 3 -:**
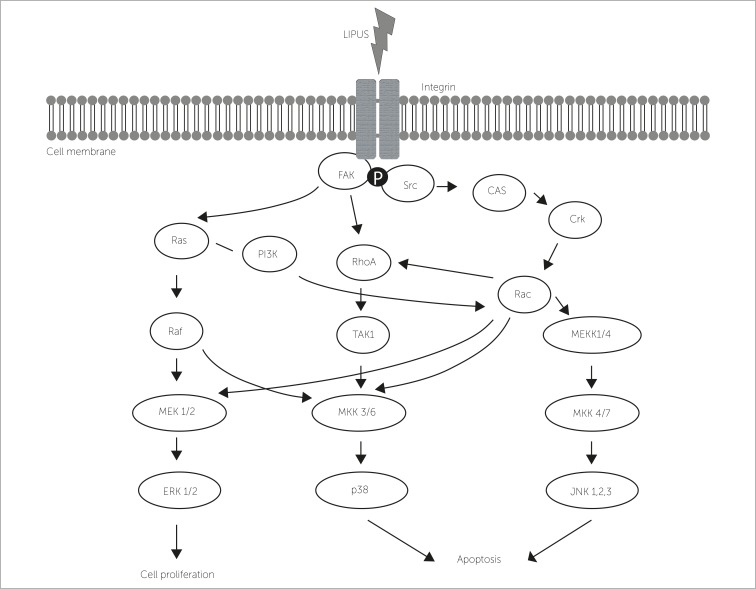
Schematic illustration of the concept of mandibular condylar cartilage degradation. Functional overloading can facilitate hypoxia in the TMJ and mediate the destructive processes associated with osteoarthrosis as an autocrine factor. VEGF induction in OA-cartilage by functional overloading is linked to activation of HIF-1, leading to hypxia in the joint tissue. Furthermore, VEGF regulates the production of MMPs and TIMPs which are among the effectors of extracellular matrix remodeling. Overloading also causes collapse of joint lubrication as a result of HA degradation by free radicals.

## Firstly, it is necessary to thank for this opportunity and congratulate Dental Press Journal of Orthodontics for the efforts on providing every issue with excellent interviews that always bring relevant and updated information. Professor Tanaka, evolution on genetic science has made possible the understanding and treatment of many kinds of disease. Based on that, genetic knowledge has also been addressed for the treatment of temporomandibular disorders (TMD). Could you please comment about the current status of genetic approach for TMD treatment? Matheus Melo Pithon

Many studies have reported that transforming growth factor (TGF)-β signals play important roles in maintaining normal status of articular cartilage, especially TGF-β1 which has been implicated in human OA. Smad3 is required to maintain articular cartilage in the quiescent state by repressing chondrocyte hypertrophic differentiation and regulating matrix molecule synthesis. Previous data indicate that genetic variation in the Smad3 gene is involved in the risk of both hip and knee OA in European populations. Similarly to other synovial joints, TMJ-OA is a degenerative disease marked by permanent cartilage destruction and extracellular matrix (ECM) loss. Genetic mouse model of TMJ-OA is deficient in two ECM proteins, biglycan and fibromodulin. The early basis for TMJ-OA arises from abnormal and accelerated chondrogenesis. TGF-β1 is a growth factor that is critical for chondrogenesis and binds to both biglycan and fibromodulin.

The genetic approach to TMD has not been developed yet, but we will focus on ECM, biglycan and fibromodulin, as novel key players in regulating chondrogenesis and ECM turnover during TMJ-OA pathology.

## Could you please briefly describe your research and results about DNA Medicine for treatment of muscle atrophic diseases? Matheus Melo Pithon

I deeply appreciate your interest in our work about treatment of muscle atrophic diseases using RNA interference. Duchenne muscular dystrophy (DMD), an X-linked recessive disorder, is the most common and severe form of childhood muscular dystrophy, which is caused by mutations in the dystrophin gene. It is a severe muscle wasting disorder that affects 1/3500 male births in humans. The effects of the disease also progressively influence oral function. A high prevalence of oral dysfunction with malocclusion has been noted in DMD patients, including lower bite force, dysphagia, severe open bite and posterior crossbite with a steep mandibular plane, all of which appear to be strongly related to the involvement of masticatory muscles in the disease. To date, there is no effective treatment for muscular dystrophy, although gene therapy could be a valuable approach to treating the disease. Growing evidence suggests that small-interfering RNA (siRNA) can promote gene silencing in mammalian cells without induction of interferon synthesis or nonspecific gene suppression. Recently, a number of highly specific siRNAs targeted against disease-causing or disease-promoting genes have been developed. Myostatin (Mst) is a negative regulator of skeletal muscle differentiation of which inhibition results in acceleration of muscle differentiation by satellite cell activation. Moreover, Mst knockout in mice has been reported to increase myogenesis and decrease adipogenesis. Therefore, we hypothesized that Mst inhibition by Mst-siRNA would promote myogenesis and also inhibit adipogenesis. For local administration of Mst-siRNA, atelocollagen or cationic liposome were used as a carrier of siRNA delivery. After local application of Mst-siRNA, masseter muscles of DMD model mice were enlarged, while no significant change was observed on the control side treated with scrambled-siRNA. Histological analysis showed that myofibrils of masseter muscles treated with Mst-siRNA were significantly larger than those of control. Real-time PCR analysis showed significant downregulation of Mst expression in the treated masseter. In addition, expression of myogenic transcription factors was upregulated in the Mst-siRNA-treated masseter muscle, while expression of adipogenic transcription factors was significantly downregulated. These data suggest that local administration of Mst-siRNA/carrier complex could lead to skeletal muscle hypertrophy and recovery of muscular function in muscular atrophic diseases. Therefore, local and systemic application of Mst-siRNA with a delivery carrier will be a potential tool for therapeutic use in muscular atrophic diseases in the near future.

## Patients suffering from muscle atrophic diseases are progressively seeking treatment in dental offices. How can the clinician take part in the upcoming and promising genetic therapies for TMD? Matheus Melo Pithon

This is a very difficult question to answer because the upcoming and promising genetic therapies for TMD have not been developed yet. As you know, TMD is a multifactorial disease, and neither non-invasive and invasive treatment remedies for TMD have been recognized. Once TMJ has been severely damage, as in internal derangement and bone deformation, this damage can not be repaired completely. However, should we develop a "good as new" genetic therapy for TMD, clinicians will play an important role as MD busters.

**Figure 4 -:**
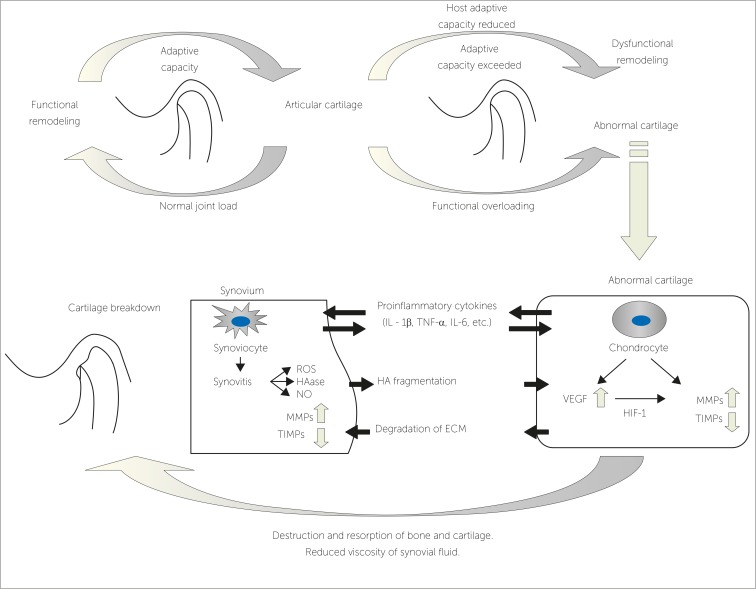
Local administration of Mst-siRNA/atelocollagen complex causes enlargement of the masseter muscle in mutant caveolin-3 transgenic (mCAV-3Tg) mice, an animal model for muscular dystrophy. A) Photographs of siRNA-treated muscles. The left muscle injected with Mst-siRNA/atelocollagen complex shows a marked increase in muscle mass compared to the right muscle injected with the control siRNA. B) Average muscle weight. The muscle weight of the Mst-siRNAtreated masseter muscle is significantly larger than that of the control muscle. C) Hematoxylin and eosin staining of the control and Mst-siRNA-treated masseter muscles. Scale bars = 50 µm. D) Average cross-sectional areas. The sectional area of fiber is significantly larger in the Mst-siRNA-treated masseter muscle than in the control. E) The ratio of the amount of myostatin mRNA for the masseter muscles. mRNA expression level in the Mst-siRNA-treated masseter muscle is significantly higher than that in the control masseter muscle. Data are expressed as means ± SD. (** P < 0.01, n = 12.).

Finally, I would like to express my sincere appreciation to all interviewers in Dental Press Journal of Orthodontics as well as their readers.
